# Which Extraction Solvents and Methods Are More Effective in Terms of Chemical Composition and Biological Activity of *Alcea* *fasciculiflora* from Turkey?

**DOI:** 10.3390/molecules27155011

**Published:** 2022-08-06

**Authors:** Refiye Beyza Ozturk, Gokhan Zengin, Kouadio Ibrahime Sinan, Domenico Montesano, Dimitrina Zheleva-Dimitrova, Reneta Gevrenova, Abdullahi Ibrahim Uba, Uğur Çakılcıoğlu, Alevcan Kaplan, Sharmeen Jugreet, Stefano Dall’Acqua, Mohamad Fawzi Mahomoodally

**Affiliations:** 1 Physiology and Biochemistry Research Laboratory, Department of Biology, Science Faculty, Selcuk University, Konya 42130, Turkey; 2 Department of Pharmacy, University of Naples Federico II, Via D. Montesano 49, 80131 Naples, Italy; 3 Department of Pharmacognosy, Faculty of Pharmacy, Medical University-Sofia, 1431 Sofia, Bulgaria; 4 Department of Molecular Biology and Genetics, Faculty of Engineering and Natural Sciences, Kadir Has University, Istanbul 34083, Turkey; 5 Pertek Sakine Genç Vocational School, Munzur University, Tunceli 62500, Turkey; 6 Sason Vocational School, Batman University, Batman 72100, Turkey; 7 Department of Health Sciences, Faculty of Medicine and Health Sciences, University of Mauritius, Réduit 80837, Mauritius; 8 Department of Pharmaceutical and Pharmacological Sciences, University of Padova, Via Marzolo 5, 35131 Padova, Italy

**Keywords:** *Alcea fasciculiflora*, phenolic, flavonoid, antioxidant, enzyme inhibition, solvents/extraction methods

## Abstract

The bioactive content, antioxidant properties, and enzyme inhibition properties of extracts of *Alcea fasciculiflora* from Turkey prepared with different solvents (water, methanol, ethyl acetate) and extraction methods (maceration, soxhlet, homogenizer assisted extraction, and ultrasound assisted extraction) were examined in this study. UHPLC-HRMS analysis detected or annotated a total of 50 compounds in *A. fasciculiflora* extracts, including 18 hydroxybenzoic and hydroxycinnamic acids, 7 Hexaric acids, 7 Coumarins, 15 Flavonoids, and 3 hydroxycinnamic acid amides. The extracts had phenolic and flavonoid levels ranging from 14.25 to 24.87 mg GAE/g and 1.68 to 25.26 mg RE/g, respectively, in the analysis. Both DPPH and ABTS tests revealed radical scavenging capabilities (between 2.63 and 35.33 mg TE/g and between 13.46 and 76.27 mg TE/g, respectively). The extracts had reducing properties (CUPRAC: 40.38–78 TE/g and FRAP: 17.51–42.58 TE/g). The extracts showed metal chelating activity (18.28–46.71 mg EDTAE/g) as well as total antioxidant capacity (phosphomolybdenum test) (0.90–2.12 mmol TE/g). DPPH, ABTS, FRAP, and metal chelating tests indicated the water extracts to be the best antioxidants, while the ethyl acetate extracts had the highest overall antioxidant capacity regardless of the extraction technique. Furthermore, anti-acetylcholinesterase activity was identified in all extracts (0.17–2.80 mg GALAE/g). The water extracts and the ultrasound-assisted ethyl acetate extract were inert against butyrylcholinesterase, but the other extracts showed anti-butyrylcholinesterase activity (1.17–5.80 mg GALAE/g). Tyrosine inhibitory action was identified in all extracts (1.79–58.93 mg KAE/g), with the most effective methanolic extracts. Only the ethyl acetate and methanolic extracts produced by maceration and homogenizer aided extraction showed glucosidase inhibition (0.11–1.11 mmol ACAE/g). These findings showed the overall bioactivity of the different extracts of *A. fasciculiflora* and provided an overview of the combination of solvent type and extraction method that could yield bioactive profile and pharmacological properties of interest and hence, could be a useful reference for future studies on this species.

## 1. Introduction

Traditional medicine has been the most economical and accessible form of therapy in the primary healthcare system, particularly in regions with limited access to modern drugs, for many years [1]. Indeed, the use of medicinal plants is an important element of traditional medicine that is deeply ingrained in the culture of people in developing nations. In addition, with the development of technology and new scientific study methodologies, a rising number of studies on phyto-active components in plants, their activities, and their curative properties are gaining prominence. The chemicals in these plants are isolated and provided in accordance with the pharmacopoeia in various pharmaceutical forms, doses, and packaging [2].

Among numerous plant families, the Malvaceae family are exceptional among leafy plants owing to their abundant content in polyprenols, which are chemotaxonomic markers. They have also been shown to possess high content of cyclopropane acids, which have not been detected in plants of other families. Amid the various species belonging to this family, *Alcea* L. (Malvaceae) is well known, having subspecies with remarkable flowering plants, they bloom with relatively large and very colourful flowers [3]. The genus *Alcea* has about 70 species worldwide and is distributed in Mediterranean and Iran-Turanian phytogeographical region [4,5]. *Alcea* is represented by 18 species in the Flora of Turkey [6].

There are numerous medicinal properties of *Alcea* members, including antioxidant, hepatoprotective, antimicrobial, antiviral, and others, but they do not include any mind-altering or psychoactive properties, possibly due to the low content of alkaloids and closely active compounds found in *Alcea*, which have no reported toxic effects [3].

Moreover, several *Alcea* species have been documented in traditional medicine. In Persian medicine for instance, *A. digitata* is employed to heal the coughs, to reduce the swelling of mucus membranes of stomach and intestines, inflations of brain, ears and eyelids, for healing wounds and to relieve pain from swellings and wens [7]. Additionally, the roots of *A.rosea* has been used in Iranian traditional medicine for a wide range of ailments, including diarrhoea, constipation, inflammation and angina [8] and is also used to treat respiratory disorders such as coughs, asthma and chronic bronchitis [9,10]. Moreover, some *Alcea* plants have been reported to be used for treating boils [11].

In addition to *Alcea* species holding a great history of folkloric medicinal uses, they have also been analysed from a scientific point of view by many researchers. While some *Alcea* species have been studied for their chemical profiles and pharmacological properties, some are still unknown in that context. Among collected scientific data, *A. setosa* is likely to be an effective antioxidant and seems to have the potential to be utilized in breast and colon cancers treatment given its preferential cytotoxicity against cancer cells [12]. Furthermore, phytochemical investigation of the alcoholic extract of the flowers of *A. rosea* led to the isolation of flavonoids, whereby kaempferol-3-*O*-[6″-(E-coumaroyl)]-*β*-d-glucopyranoside showed potent cytotoxic activity against HepG-2 cell line with high selectivity towards hepatocellular carcinoma in vitro. Dihydrokaempferol-4′-*O*-*β*-d-glucopyranoside and dihydrokaempferol displayed significant antioxidant activity and kaempferol-3-*O*-*β*-d-glucopyranoside showed an important immune stimulant activity [13].

Hence, based on promising findings on various species of *Alcea*, the aim of this paper was to investigate the bioactive contents, antioxidant, enzyme inhibition potential of the aerial part extracts of *A. fasciculiflora* from Turkey, paying particular attention to the effect of different solvents (ethyl acetate, methanol and water) and extraction methods (maceration, soxhlet, homogenizer assisted extraction, ultrasound assisted extraction) on the phytochemical yields and bioactivities of *A. fasciculiflora*.

## 2. Results and Discussion

### 2.1. Chemical Profiling

In the present study, we selected three solvents for each extraction methods. It has been shown in the literature that polar solvents are more effective (high level of total phenolic and flavonoid contents) when dealing with *Alcea* species [14,15,16,17,18]. Thus, ethyl acetate and methanol were chosen organic solvents to observe differences between polar solvents. As a traditional purpose, water was used for each extraction methods.

The extracts showed varying total phenolic and flavonoid contents in the tested extracts. For instance, the total phenolic content ranged from 14.25–24.87 mg GAE/g, while the total flavonoid content ranged from 1.68–25.26 mg RE/g. In particular, the methanolic extracts obtained from SOX, HAE and UAE showed higher TFC compared to the other extracts (Table 1). In a recent paper by Taskın et al. [18], the levels of total bioactive compounds in *A. disecta* extracts obtained from different solvents in two extraction technique (Soxhlet and maceration) were reported. In their study, the total phenolic content was found to be 3.3–5.8 mg GAE/g (in Soxhlet extracts) and 4.4–12.8 mg GAE/g (in maceration extracts), which was lower than in the presented work. In addition, Ertas et al. [4] investigated the effect of different solvent extracts (petroleum ether, acetone, methanol and water) of two *Alcea* species (*A. pallida* and *A. apterocarpa*) on the concentrations of total bioactive compounds and generally, the tested acetone extracts contained higher level when compared with other solvents. However, the ethanol extract of *A. apterocarpa* seeds obtained by maceration technique was reported as the richest extract [15]. In another study conducted by Azadeh et al. [16], the total phenolic and flavonoid contents depened on the parts (flower and herb) of four *Alcea* species (*A. koelzii*, *A. arbelensis*, *A. aucheri* var. *lobata* and *A. aucheri* var. *aucheri*) and in general the flower extracts had more concentrations of the compounds than herb. Zakizadeh et al. [19] reported that the total phenolic and flavonoid contents of the methanol extract of *A. hyrcana* flowers was 48.1 mg GAE/g and 24.3 mg quercetin equivalent (QE)/g, respectively. the higher levels of total phenols and flavonoids were also found in the flower extract of *A. pallida* compared to the stem extract [17]. These observations showed that the total content of bioactive compounds in members of the genus *Alcea* depended on plant parts, extraction methods and solvents. However, no data were found detailed chemical characterization of *A. fasciculiflora* extracts by further chromatographic techniques.

In the present study, secondary metabolites dereplication in the tested *A. fasciculiflora* extracts was performed by UHPLC-HRMS. Based on elemental composition, accurate mass, fragmentation pathways in MS/MS, and comparisons to standard references and/or literature data, a total of 50 metabolites including 9 hydroxybenzoic and hydroxycinnamic acids and derivatives with 9 glycosides, 7 hexaric acids, 7 coumarins, 15 flavonoids, and 3 hydroxycinnamic acid amides were identified or tentatively annotated (Table 2).

### 2.2. Hydroxybenzoic, Hydroxycinnamic Acids and Derivatives

Compounds **3**, **8**, **12**, **16**, and **18** were a unambiguously identified in the studied extracts by comparison with standard references (Table 2). Phenolic acids hexosides (1, 2, 5, 6, 7, 10, 11, 13) were witnessed by the common loss of hexose unit of *m*/*z* 162.05. Compound **9** [M-H]^−^ at *m*/*z* 153.055, gave fragment ion at *m*/*z* 137.024 [M-H-CH_4_]^−^, and a base peak at *m*/*z* 123.044 [M-H-HCHO]^−^. Thus, 9 could be related to vanillic alcohol. Compound **4** differed from 9 by 162.05 Da and revealed the same fragmentation pathway and could be ascribed to vanillic alcohol O-hexoside (Table 2).

### 2.3. Hexaric Acid Derivatives

Hexaric acid derivatives can be identified in the extracted ion chromatograms by the common ion at *m*/*z* 209.030 [hexaric acid (HA)-H]^−^ (C_6_H_9_O_8_) in MS/MS spectra, accompanied by a series of fragment ions resulting from the neutral losses at *m*/*z* 191.019 [HA-H-H_2_O]^−^, 147.029 [HA-H-H_2_O-CO_2_]^−^, 129.018 [HA-H-2H_2_O-CO_2_]^−^, 111.007 [HA-H-3H_2_O-CO_2_]^−^, 85.028 [HA-H-2H_2_O-2CO_2_]^−^ (Table 2) [20]. Compounds **19**–**21** shared the same [M-H]^−^ at *m*/*z* 355.067 (C_15_H_15_O_10_). They produced the indicative fragment ion at *m*/*z* 209 resulting from the loss of 146 Da. *p*-Coumaroyl moiety was suggested by the low abundant ions at *m*/*z* 163.039 [*p*-coumaric acid (*p*-CoA)-H]^−^ and 119.049 [*p*-CoA-H-CO_2_]^−^. Compounds **19**–**21** were ascribed as isomeric *p*-coumaroylhexaric acids. In the same manner, 22–24 ([M-H]^−^ at *m*/*z* 385.078, C_16_H_17_O_11_) were assigned to feruloylhexaric acids isomers as indicated by the common fragment ions at *m*/*z* 193.050 [ferulic acid (FA)-H]^−^ and 134.036 [FA-H-CO_2_-CH_3_^•^]^−^.

### 2.4. Coumarins

Peak 31 gave a base peak at *m*/*z* 117.033 [M-H-CO]^−^, and fragment at *m*/*z* 89.038 corresponding to the subsequent losses of two carbonyl groups, and could be attributed to coumarin. Similarly, 29 [M-H]^−^ at *m*/*z* 161.023 gave a base peak at *m*/*z* 133.028, due to the loss of CO group, and fragment ions at *m*/*z* 115.018 [M-H-CO-H_2_O]^−^ and *m*/*z* 105.033 [M-H-2CO]^−^. 29 and 27 differ from 31 by one and two OH groups and could be related to umbelliferone and aesculetin, respectively (Table 2) [21]. In the fragmentation pattern of 30 and 32, the base peaks at *m*/*z* 192.005 [M-H-CH_3_^•^]^−^ and 176.011 [M-H-CH_3_^•^]^−^ corresponded to the loss of CH_3_^•^ radical. Thus, 30 and 32 were tentatively annotated as fraxetin and scopoletin, respectively (Table 2). Compounds **26** and **28** differed from 27 (aesculetin) and 30 (fraxetin), by 162.05 Da, and could be ascribed to their hexosides aesculin and fraxin, respectively (Table 2).

### 2.5. Flavonoids

A variety of flavonoid-*O*-glycosides including 2 myricetin, 1 isorhamnetin, 1 patuletin, 2 quercetin, and 3 kaempferol derivatives were identified in the studied extracts. Hexosyl, hexuronyl, and rutinosyl moieties were witnessed by the neutral losses of 162.05, 176.03, and 308.11 Da. The flavonoid aglycons luteolin (41), quercetin (42), and naringenin (45) were deduced from the Retro-Diels-Alder (RDA) rearrangements ^1,3^A^−^, ^0,4^A^−^, ^1,2^A^−^, ^1,3^B^−^ and ^1,2^B^−^ (Table 2). Compounds **37**, **38**, **39**, **41**, **42**, and **43** were identified by comparison with reference standards (Table 2).

### 2.6. Cinnamic Acid Amides

Cinnamic acid amides 48–50 were dereplicated in (+) ESI-MS/MS (Table 2). Two tyramine amides (48, 49) were discernable by the common loss of a tyramine moiety (137.084 Da, C_8_H_11_ON) at *m*/*z* 147.044 [*p*-CoA + H-H_2_O]^+^ (48) and 177.055 [FA + H-H_2_O]^+^ (49) supported by *m*/*z* 121.065 [tyramin + H-NH_3_]^+^ [22]. Accordingly, N-*p*-coumaroyl and N-feruloyl-tyramine were evidenced in the extracts. N-feruloyl 3-methoxytyramine was deduced from [M + H]^+^ at *m*/*z* 344.149 (C_19_H_22_O_5_N) and ferulic acid-derived fragment ions at *m*/*z* 177.054 [FA + H-H_2_O]^+^, 145.028 [FA + H-H_2_O-CH_3_OH]^+^ and 117.034 [FA + H-CO-CH_3_OH]^+^ (Table 2).

### 2.7. Chemometrics Analysis

**Figure 1:**
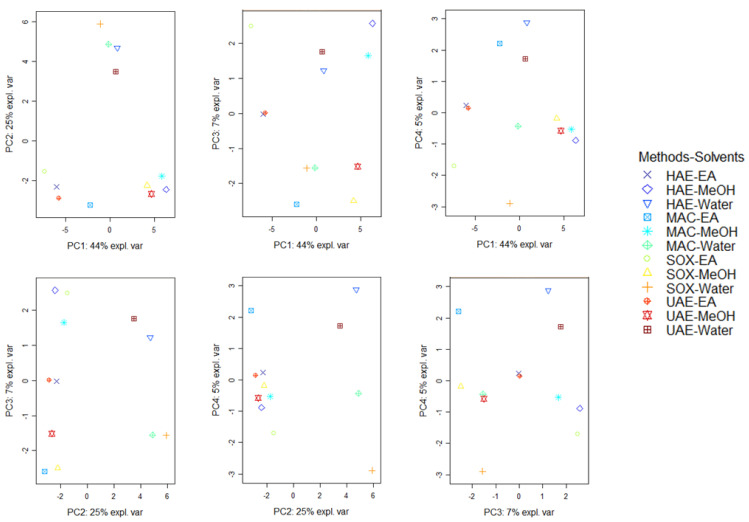
Score plots of Principal component analysis on the secondary metabolites of *Alcea fasciculiflora*.

PCA is a statistical method for identifying differences and similarities among samples and elucidating the factors that contribute to these differences and similarities. The analysis is the first on the chemical profile and biological properties of *A. fasciculiflora* extracts obtained with different solvents from different extraction methods. Thus, PCA could provide a scientific starting point for selecting the most effective solvents and methods for future applications using this plant. Using loading plots and a supplementary set of scores, this approach identifies the most significant patterns in the data set. First, PCA may be used to detect relationships between samples, and second, it can be used to investigate how many principal components (a linear combination of starting variables) are necessary to summarize the major proportion of variance with a minimum loss of information [23]. A summary of PCA results based on secondary metabolites data is shown in Figure 1. Referring to a factor loadings analysis (Table S1) The first four principal components (PCs) retained about % of the total variability, about 44% in the first principal component (PC1), 25% in the second (PC2), 7% in the third (PC3) and 5% in the fourth (PC4). PC1 differentiate the samples mainly according to a large number of secondary metabolites (about thirty secondary metabolites) (Figure S1). PC2 separated the samples, predominantly, based on approximatively ten secondary metabolites. PC3 and PC4 distinguished the samples in depending on very few secondary metabolites.

**Figure 2:**
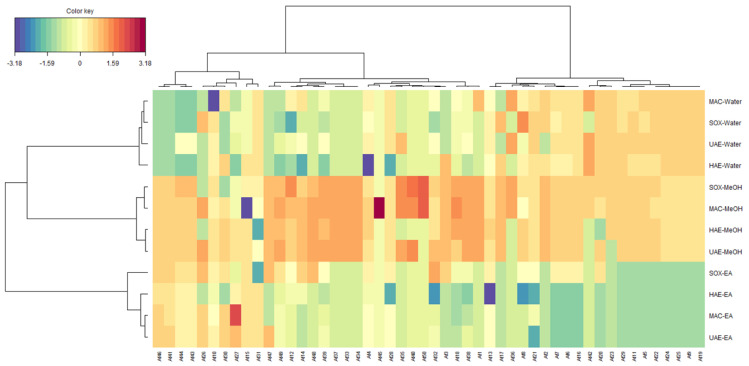
Clustered image maps on the Secondary metabolites in *Alcea fasciculiflora* extracts. Red colour: Highest concentration. Blue Colour: Lowest concentration. For compounds numbers refer to Table 1.

Once the representative PCs were determined, on the basis of scores plots, a comparison of the positioning of samples to each other was performed (Figure 1). In the light of all the score plot, it can be observed in the first score plot (PC1 vs. PC2) that the samples were grouped together regardless the type of extractive solvent, pointing out that the solvent had a greater effect on secondary metabolites extraction than the extractive methods. This finding was confirmed by the clustered image maps (CIM) analysis carried out using the coordinates of the samples on the first four PCs of the PCA (Figure 2). By observing the heatmap, it is evident that methanol extracts were the richest in secondary metabolites, following by the water extracts (richest in secondary metabolites within the group C) and ethyl acetate extracts (richest in secondary metabolites belonging to the group A). Several studies have demonstrated the effect of the extraction solvent on the secondary metabolites of medicinal plants. Thus, according to Mohammad Salamatullah, et al. [24], the concentration of secondary metabolites can be directly linked to the solvent proprieties i.e., hydrophilic and lipophilic solvents and their respective polarity.

### 2.8. Antioxidant Properties

Plant materials (such as herbs, seeds, spices, fruits and vegetables) are considered natural sources of antioxidants. Antioxidants can protect cells in a number of ways, including converting ROS into non-radical species, preventing ROS from initiating an auto-oxidative chain reaction, and reducing oxygen levels in cells [25]. Polyphenols, which exhibit a wide range of structural, functional and biological properties, are the most important plant antioxidants [26]. In the present study, the extracts were found to possess radical scavenging ability, demonstrated in both DPPH and ABTS assays (2.63–35.33 mg TE/g and 13.46–76.27 mg TE/g) (Table 3). Interestingly, in both assays, the water extracts displayed the highest scavenging capacity, followed by the methanolic and ethyl acetate extracts. The extracts also possessed reducing capacity in CUPRAC and FRAP assays (40.38–78.32 mg TE/g and 17.51–42.58 mg TE/g) (Table 2). Among the extracts that displayed the highest potency in CUPRAC assay were the methanolic extracts obtained by HAE and MAC and the ethyl acetate extracts obtained by SOX and UAE. On the other hand, the water extracts were found to have the highest reducing activity in FRAP assay. Metal chelating activity was also noted by the extracts in the range of 18.28–46.71 mg EDTAE/g. Remarkably, the same pattern was seen with the metal chelating assay as for the scavenging and FRAP assays, whereby the water extracts demonstrated the highest activity. Total antioxidant capacity of the extracts was also revealed by the phosphomolybdenum assay, ranging from 0.90–2.12 mmol TE/g. However, unlike most of the antioxidant assays, the ethyl acetate extracts presented the highest total antioxidant capacity, irrespective of the extraction method used. Antioxidant properties of members of the genus *Alcea* have been reported in the literature by several authors. For example, in a study performed by Taskın et al. [18], the antioxidant properties of different extracts of *A. dissecta* were investigated by several antioxidant assays (DPPH, ABTS, FRAP and CUPRAC) and the Soxhlet extracts exhibited greater potentials, except for ABTS. In another study, the acetone and water extracts of *A. pallida* and *A. apterocarpa* showed the strongest ABTS and DPPH scavenging abilities [4]. Azadeh et al. [16] reported IC_50_ values for DPPH scavenging abilities of four *Alcea* species and the ability depended on both plant parts and species (4649–263.47 µg/mL). Anlas et [15] found that the extracts of *A. apterocarpa* obtained from Soxhlet had stronger DPPH scavenging abilities compared to the extracts obtained by maceration. In general, this observation was consistent with our presented results. Our findings are in line with results reported by Alhage and Elbitar [14], who found that the methanol and water extracts of *A. setosa* showed greater DPPH scavenging abilities than dichloromethane extracts. In the study of Zakizadeh, Nabavi, Nabavi and Ebrahimzadeh [19], the *A. hyrcana* leaf extract exhibited strong ferrous chelating activity, nitric oxide radical scavenging and better reducing power activity than other extracts, while the seed extract showed high scavenging activity against free radicals, including both the hydrogen peroxide and DPPH radicals. The seed extract also showed significant higher total phenol, while the leaf extract had higher flavonoids contents than other parts. In light of the observations, polar solvents including acetone, methanol, or water might be more useful for the members of the *Alcea* genus to extract more secondary metabolites and provide high antioxidant properties.

**Figure 3:**
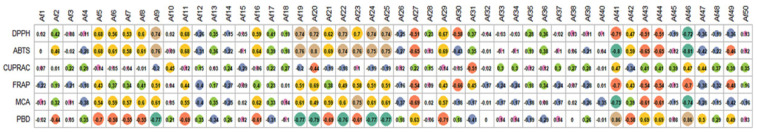
Relationship between secondary metabolites and antioxidant activities of *Alcea fasciculiflora.* For compounds numbers refer to Table 1. The correlation is considered positive and statistically significant (r > 0.7).

In the multivariate analysis, several secondary metabolites seemed to be involved in the observed antioxidant activity, given that a positive and significant correlation (r ≥ 0.7) was observed between various secondary metabolites and the different antioxidant activities (ABTS, DPPH, MCA and PPBD) (Figure 3). The relevant secondary metabolites include the following: p-coumaroylhexaric acid isomer (Af9), luteolin (Af41), apigenin (Af46), p-coumaroylhexaric acid (Af19), p-coumaroylhexaric acid isomer (Af20), p-coumaroylhexaric acid isomer (Af21), feruloylhexaric acid (Af22), feruloylhexaric acid isomer (Af23), feruloylhexaric acid isomer (Af24), feruloylhexaric acid isomer (Af25).

### 2.9. Enzyme Inhibition Effects

**Figure 4:**
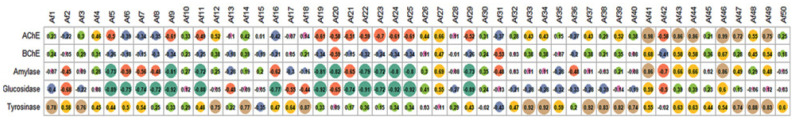
Relationship between secondary metabolites and enzyme inhibitory activities of *Alcea fasciculiflora.* For compounds numbers refer to Table 1. The correlation is considered positive and statistically significant (r > 0.7).

Deterioration in cognitive abilities, including memory loss, learning difficulties, and the inability to do simple everyday tasks, are hallmarks of Alzheimer’s disease (AD), the most common neurodegenerative disorder. In clinical studies with AD patients, only one class of drugs has been extensively evaluated and authorized by the US Food and Drug Administration (FDA). Alzheimer’s disease (AD) individuals who use cholinesterase inhibitors noticeably improve their cognitive function, but the benefit is limited [27]. Plant extracts and compounds isolated from plants have indeed been in the forefront in the search for novel cholinesterase inhibitors during the past decades [28,29]. All extracts were found to possess anti-AChE activity (0.17–2.80 mg GALAE/g). However, the least anti-AChE activity was noted for the water extracts irrespective of their extraction methods (0.17–0.39 mg GALAE/g). On the other hand, while all the water extracts and the ethyl acetate extract obtained by UAE were inactive against BChE, the other extracts showed anti-BChE activity in the range of 1.17–5.80 mg GALAE/g (Table 4). In the literature, few studies have been reported the enzyme inhibitory properties of members of the *Alcea* genus. In the study of Ertas, Boga, Gazioglu, Yesil, Hasimi, Ozaslan, Yilmaz and Kaplan [4], at 200 μg/mL concentration, among the tested extracts, the *A. pallida* acetone extract showed the highest inhibition against BChE. However, the best BChE inhibitory effect was found in the methanol extract of *A. apterocarpa*. The tested water extracts showed a weaker ability to inhibit BChE and this finding was consistent with our results for *Alcea fasciculiflora*. In another study, AChE inhibition abilities of the methanol extracts of the aerial organs (flowers, stems, leaves) of *A. kurdica* (Schlecht) were also investigated by Mohammadi, et al. [30]. In their study, the methanol extract from flower organ of *A. kurdica*, at 2 mg/mL concentration, inhibited the enzyme activity with 63.45% inhibition (IC_50_ value: 0.114 mg/mL). The results were consistent with our results, where the ethylacetate and methanol extracts were more stronger inhibitors on AChE and BChE when compared to water extracts. To provide more information on the relationship between chemical composition and anticholinesterase activity, we performed a correlation analysis. As can be seen in Figure 4, AChE activity was significantly positively linked to Luteolin (Af41), tiliroside (Af43), kaempferol-O-p-coumaroyl-O-hexoside (Af44), apigenin (Af46), kaempferol (Af47), N-feruloyl tyramine (Af48).

Tyrosinase is a multi-copper enzyme that plays a critical role in melanogenesis and enzymatic browning in a variety different organisms. This enzyme is a popular target for depigmentation chemicals in the cosmetics and pharmaceutical industries, and as anti-browning agents in the food and agricultural industries [31]. There are a wealth of active chemicals in plants and plant extracts that may be used to inhibit tyrosinase and treat skin conditions caused by melanin hyperpigmentation at a low cost and in large quantities [32]. In this study, all the studied extracts were found to exhibit tyrosinase inhibitory effect (1.79–58.93 mg KAE/g), although the methanolic extracts were revealed to be the most potent inhibitors of tyrosinase (Table 4). This might be related the higher TFC in the methanolic extracts. Likewise, significant positive correlation among tyrosinase and a dozen secondary metabolites was observed (Figure 4). The anti-tyrosinase activity of some of these molecules including caffeic acid, isoquercitrin has already been demonstrated [33,34,35]. In the literature, just one paper regarding tyrosinase inhibitory properties of members of the genus *Alcea* was observed. In the study conducted by Namjoyan, et al. [36], the hydroalcholic extract of *A. rosea* inhibited tyrosinase ability with IC_50_: 0.38 mg/mL.

The extracts were also tested for their antidiabetic properties by inhibiting the enzymes α-glucosidase and α-amylase, which are involved in the digestion of carbohydrates. Type 2 diabetics may benefit from slowing carbohydrate digestion by modulating the activity of two hydrolyzing enzymes, α-glucosidase and α-amylase, to prevent postprandial hyperglycemia [37].

In this study, all extracts were found to act as amylase inhibitors (0.11–1.11 mmol ACAE/g), while glucosidase inhibition was displayed by only six of the studied extracts, that is, the ethyl acetate and methanolic extracts obtained by MAC and HAE, including the ethyl acetate extracts obtained by SOX and UAE. The more polar extracts (water and methanol) were observed to be inactive against glucosidase possibly due to the absence of particular compounds acting against the enzyme. On the other hand, significant positive correlation was observed between amylase and two secondary metabolites including luteolin (Af41), apigenin (Af46) (Figure 4). Luteolin has been reported to have the potential to control and prevent diabetes by being included into starch-based food to retard starch digestion [38]. Similarly, it was demonstrated that apigenin and luteolin potently inhibited starch-hydrolytic action of porcine prancreatic aalpha-amylase, and the percent of inhibition were 21 and 61% [39]. As far as we know, no previous research has investigated on amylase and glucosidase inhibitory effects of member of the genus *Alcea*. These results provide a first scientific starting point and evidence that members of the genus *Alcea* may have great potential to produce antidiabetic formulations.

### 2.10. Data Mining

**Figure 5:**
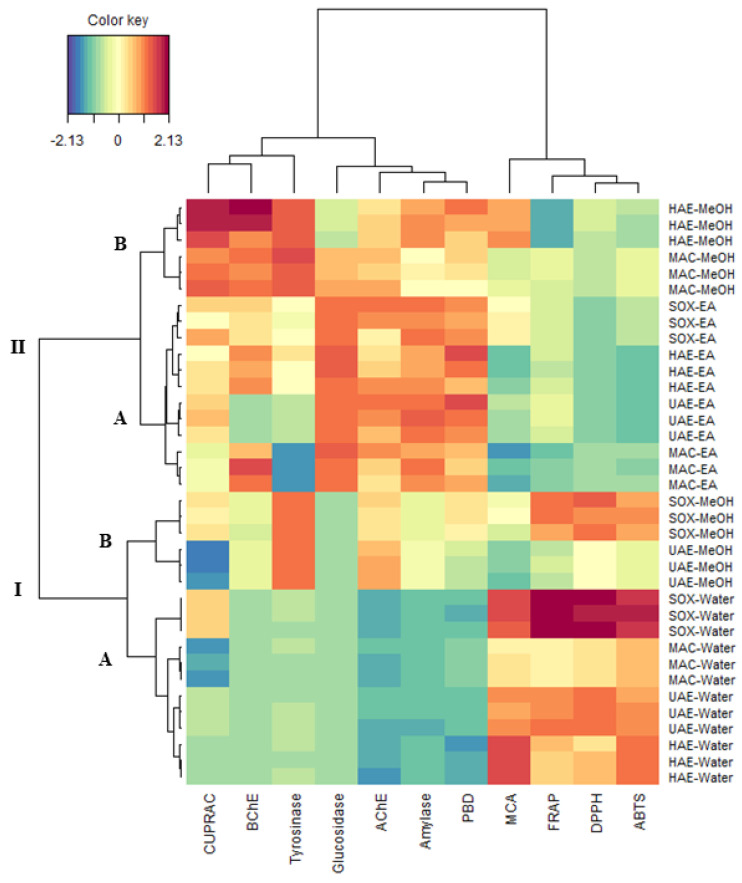
Clustered image maps on the biological activities of *Alcea fasciculiflora* extracts. Red colour: Highest activity. Blue Colour: Lowest activity.

As a complementary analysis to one-way ANOVA analysis, a clustered image maps (CIM) analysis carried out to explore the organization of samples in clusters. The result of CIM is usually presented in a 2-dimensional plot which shows the organization of samples in groups and discloses the variables characterizing each group. Figure 5 shows the representation of CIM analysis, where antioxidant and enzyme inhibitory activities were used as variables. Overall, the samples could be formed into two main clusters and four sub-clusters. The sub-cluster IA comprised of all water extracts which exhibited the highest ABTS, DPPH, FRAP and MCA activities. The sub-cluster IB included two extracts (SOX-MeOH and UAE-MeOH). The sub-cluster IIA was represented by all ethyl acetate extracts which showed the best anti-glucosidase, anti-amylase, anti-AChE and phosphomolybdenum properties. The sub-cluster IIB contained two extracts (MAC-MeOH and HAE-MeOH) which had higher anti-tyrosinase, anti-BChE and CUPRAC activity. In the view of this result, we note that the solvent influenced considerably the biological activities than the extractive methods. However, regarding the methanolic extracts, we observe a separation of the two cold extraction methods (MAC and HAE) from the two hot extraction methods (SOX and Infusion). This finding highlights the effect of heat on the biological activity of *Alcea fasciculiflora*.

### 2.11. Molecular Docking

**Figure 6:**
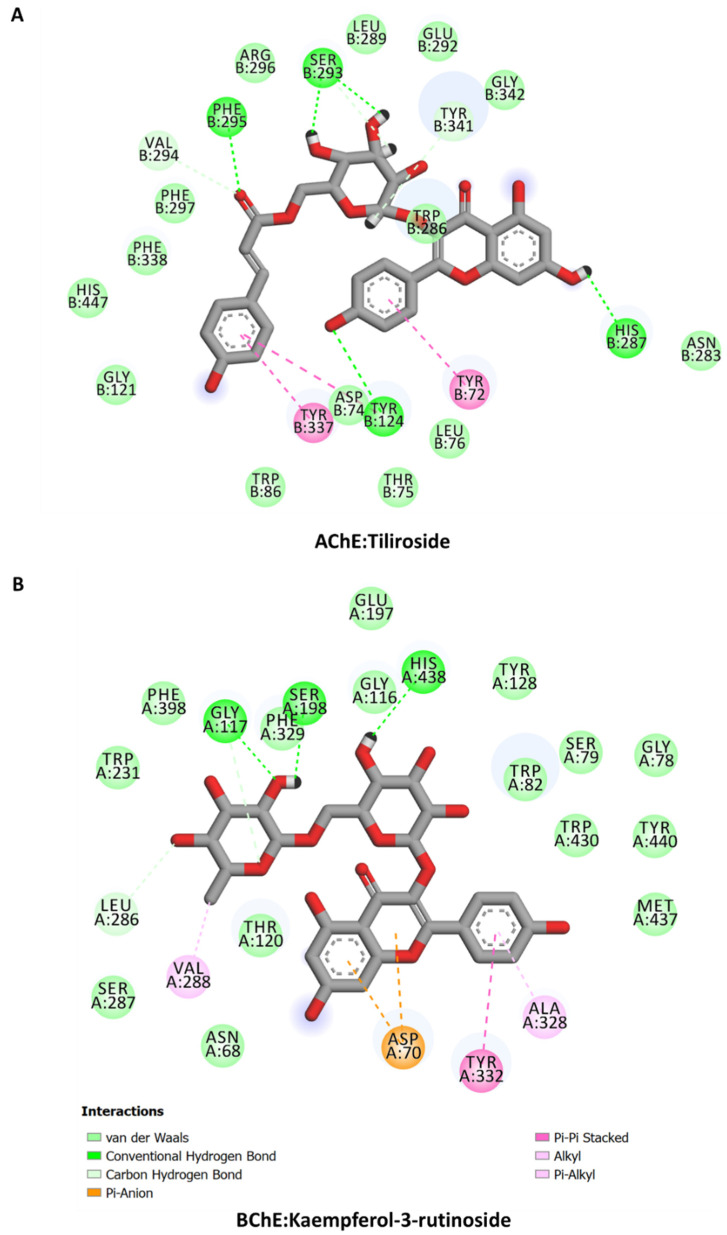
Protein-ligand interactions: (**A**) AChE and tiliroside, (**B**) BChE and Kaempferol-3-rutinoside.

**Figure 7:**
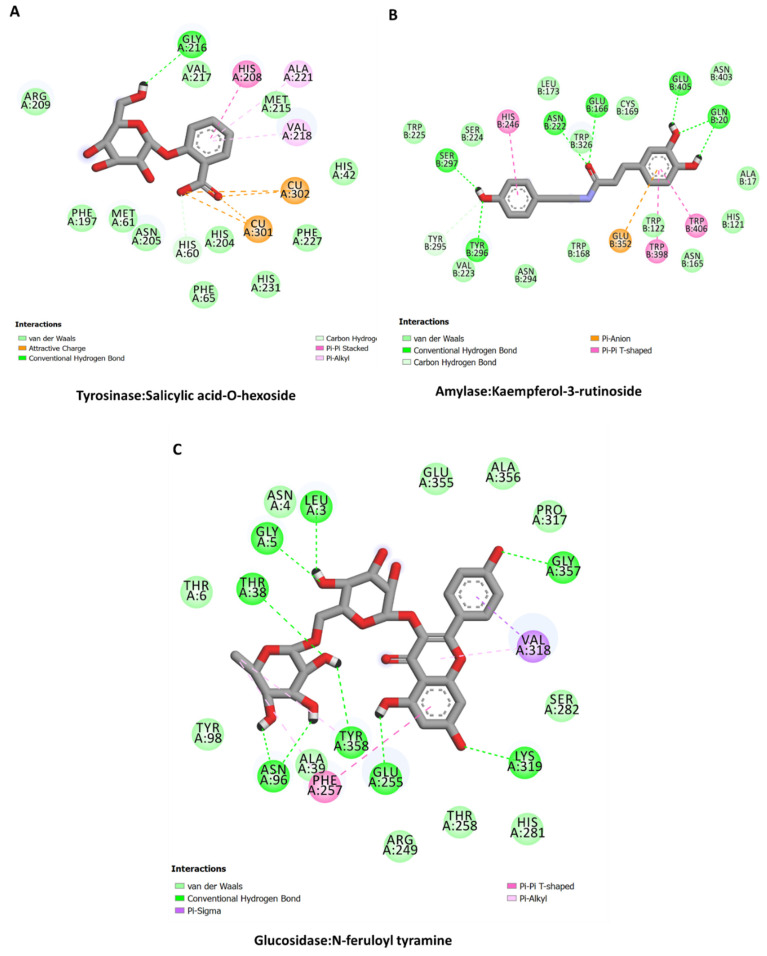
Protein-ligand interactions: (**A**) tyrosinase and salicylic acid-O-hexoside, (**B**) amylase and Kaempferol-3-rutinoside, and (**C**) glucosidase and N-feruloyl tyramine.

The calculated binding energy scores of bioactive constituents from Secondary metabolites in *Alcea fasciculiflora* extracts are presented in Table 5. All the studied compounds show potential binding to these protein targets, with good binding mode and varying binding energy scores based on different interactions. The interactions of some selected compounds are shown in Figure 6 and Figure 7. Tiliroside and Kaempferol-3-rutinoside displayed similar binding mode against AChE and BChE, respectively, forming multiple H-bonds, a couple of hydrophobic and π-π stacked, and several van der Waals interactions all over the catalytic channels of the enzymes (Figure 6). The salicylic acid-O-hexoside is completely buried in the catalytic pocket of tyrosinase. The carboxyl group of the compound formed ionic interactions with the tyrosinase two active site copper ions. Other interactions formed include a H-bond, a couple of hydrophobic interactions, π-π stacked, and multiple van der Waals interaction throughout the channel (Figure 7A). Like in the case of AChE and BChE, the major contributors to the interaction of kaempferol-3-rutinoside and N-feruloyl tyramine with amylase (Figure 7B) and glucosidase (Figure 7C), respectively, are H-bonds. Multiple H-bonds along with other weaker interactions, including van der Waals interactions enabled the compounds to firmly bind to these proteins.

**Figure 8:**
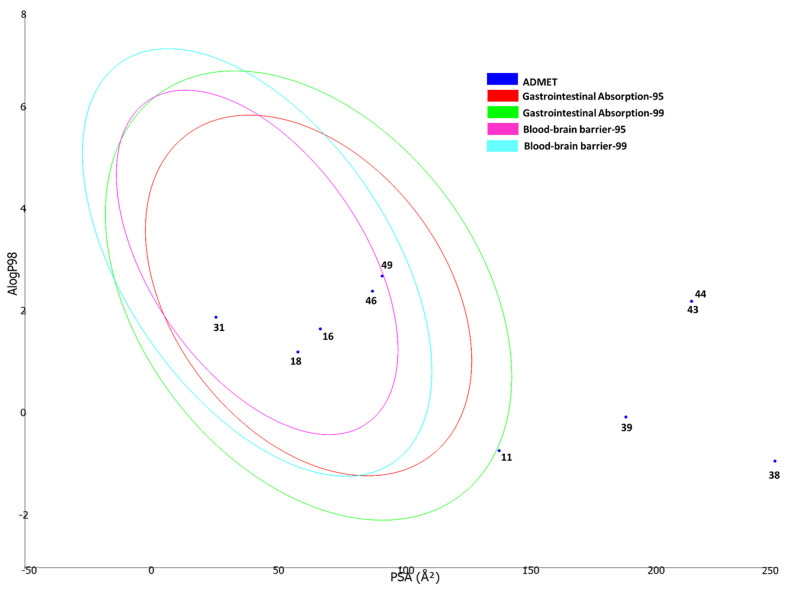
ADMET properties of the bioactive constituents from *Alcea fasciculiflora* predicted using Biovia DS ADMET prediction module. The four ellipses define regions where well absorbed compounds are expected to be located: for gastrointestinal absorption at 95 and 99% (red and green) confidence levels, and for blood-brain barrier penetration (BBB) at 95 and 99% (magenta and aqua) confidence levels. All compounds are shown according to their serial number in Table 2.

Furthermore, to predict the ADMET properties of each compound, a plot of logarithm of octanol-water partition coefficient (LogP) versus polar surface area (PSA) of each compound was generated using the ADMET prediction module in Biovia DS. There are four ellipses enclosing regions where well-absorbed compounds are expected to be located: for gastrointestinal absorption at 95 and 99% (red and green) confidence levels, and for blood-brain barrier (BBB) penetration at 95 and 99% (magenta and aqua) confidence levels. Compounds with smaller size and low polarity were predicted to fall within all or any of the four ellipses, whereas compounds with large size and high polarity were found to be outside the ellipses, and hence are associated with low absorption and low BBB penetration probability (Figure 8).

## 3. Materials and Methods

### 3.1. Plant Materials and Preparation of Extracts

*Alcea fasciculiflora* samples were collected in the city of Diyarbakır (between Egil and Dicle road, 4.5 km, 960 m), in the 2021 summer season (June). The plants were confirmed by one co-author (Dr. Ugur Cakilcioglu) in Munzur University and one voucher specimen (AK-2021/22) has been deposited in Munzur University. The plant samples (aerial parts) were dried in the shade at room temperature for approximately one week. The samples were then pulverized using a mill, and they were placed in a dark environment.

In the present study, four extraction methods (maceration (MAC), soxhlet (SOX), homogenizer-assisted (HAE) and ultrasound-assisted (UAE)) were performed using three solvents (ethyl acetate (EA), methanol (MeOH) and water). The extraction procedures are summarized in the below. The solid-solvent ratio was 1/20 in all extraction methods.

Maceration (MAC): The plant materials (10 g) were stirred with 200 mL of the solvents at the room temperature for 24 h in a shaking device.

Soxhlet (SOX): The plant materials (10 g) were extracts with 200 mL of solvents in a Soxhlet apparatus for 6 h. Regarding the water extract, the plant materials (10 g) were kept with 200 mL of boiled water for 15 min.

Homogenizer-assisted extraction (HAE): The plant materials (10 g) were extracted with 200 mL of the solvents in one ultra-turrax (6000× *g*) for 5 min.

Ultrasound-assisted extraction (UAE): The plant materials (10 g) were extracted with 200 mL of the solvents in one ultrasound bath at room temperature for 30 min.

After the extraction procedures, all extracts were filtered using Whatman No. 1 filter paper in Büchner flask under vacuum. The solvents were removed using rotary-evaporator. Regarding water extracts, the extracts were lyophilized for 48 h at −85 °C. All extracts were stored at 4 °C until analysis.

### 3.2. Total Phenolic and Flavonoid Content

Folin-Ciocalteu and AlCl3 assays were used to determine the total phenolic and flavonoid contents, respectively [40]. For respective assays, results were expressed as gallic acid equivalents (mg GAEs/g dry extract) and rutin equivalents (mg REs/g dry extract).

### 3.3. Ultra-High-Performance Liquid Chromatography Coupled with Hybrid Quadrupol-Orbitrap High Resolution Mass Spectrometry (UHPLC-HRMS)

The UHPLC separation was performed using Kromasil EternityXT C18 (1.8 µm, 2.1 × 100 mm) column maintained at 40 °C. The mobile phase consisted of A (0.1% formic acid) and B (0.1% formic acid in AcN). The run time was 33 min. The gradient was as follows: 5% B (0–1 min), 30% B (2–20 min), 50% B (21–25 min) 70% B (26–30 min), 95% B (31–33 min), then equilibrated to 5% B over 4 min. The analyses were performed on a Q Exactive Plus mass spectrometer (ThermoFisher Scientific, Inc., Waltham, MA, USA) with a heated electrospray ionization (HESI-II) probe (ThermoScientific, Waltham, MA, USA). The tune parameters were spray voltage 2.5 kV (−) and 3.5 kV (+), auxiliary gas flow rate 12, sheath gas flow rate 38, capillary temperature 320 °C, spare gas flow rate 0, probe heater temperature 320 °C and S-lens RF level 50. FS-MS spectra range from 100 to 1000, in negative and positive ionization modes and resolution of 70,000. The automatic gain control (AGC) target 1 × 10^6^, number of scan ranges 1, maximum ion time (IT) 50 ms. For DD-MS^2^, the parameters included resolution 17,500, microscans 1, AGC target 1 × 10^5^, maximum IT 50 ms, MSX count 1, Top5, isolation window 2.0 *m*/*z*, stepped normalized collision energy (NCE) 10, 30, 60. The data acquisition and processing were performed with Xcalibur 4.2 software (ThermoScientific, Waltham, MA, USA). MZmine software version 2 (MZmine Development Team, MA, USA) was applied to processing of raw UHPLC–HRMS files of the studied extracts.

### 3.4. Antioxidant Assays

Antioxidant assays were carried out according to previously reported methodologies [41,42]. The antioxidant potential was expressed as: mg Trolox equivalents (TE)/g extract in 2,2-diphenyl-1-picrylhydrazyl (DPPH) and 2,2′-azino-bis(3-ethylbenzothiazoline-6-sulfonic acid) (ABTS) radical scavenging, cupric reducing antioxidant capacity (CUPRAC) and ferric reducing antioxidant power (FRAP) tests, mmol TE/g extract in phosphomolybdenum assay (PDA) and mg ethylenediaminetetraacetic acid equivalents (EDTAE)/g extract in metal chelating assay (MCA).

### 3.5. Enzyme Inhibitory Assays

The enzyme inhibitory assays were carried out according to previously reported methodologies [41,42]. The acetylcholinesterase (AChE) and butyrylcholinesterase (BChE) inhibition was expressed as mg galanthamine equivalents (GALAE)/g extract; tyrosinase inhibition was expressed as mg kojic acid equivalents KAE/g extract; amylase and glucosidase inhibition was expressed as mmol acarbose equivalents (ACAE)/g extract.

### 3.6. Molecular Modeling

The following crystal structures of the target enzymes were retrieved from the protein data bank (https://www.rcsb.org/, accessed on 1 June 2022): AChE (PDB ID: 6O52) [43], BChE (PDB ID: 6EQP) [44], tyrosinase (PDB ID: 6QXD) [45] amylase (PDB ID: 6TP0) [46], and glucosidase (PDB ID: 7KBJ) [47]. They were prepared at physiological pH of 7.4 using Biovia Discovery Studio (DS) (Dassault Systèmes Biovia Software Inc., San Diego, CA, USA). In the protein preparation, water molecules were removed, hydrogen atoms were added, bond orders were corrected, and missing atoms were added. The 3D structure of each study ligand was downloaded from PubChem database (https://pubchem.ncbi.nlm.nih.gov/ accessed on 1 June 2022), and its geometry was optimized using “lig prep” toolkit in Biovia DS (Dassault Systèmes Biovia Software Inc., 2012).

Using AutodockTools program (https://autodock.scripts.edu, accessed on 1 June 2022) [48], docking grid files were generated using the coordinates of the cocrystal ligand in each crystal structure: (AChE: X 5.01, Y 35.37, Z −8.38 Å; BChE: X 42.16, Y −17.91, Z 42.72 Å: tyrosinase: X 29.99, Y 18.21, Z 96.45 Å; amylase: X −1.54, Y −44.04, Z 22.63 Å; glucosidase X −13.77, Y 24.04, Z 12.35 Å). For ligand conformational search, the Lamarckian genetic algorithm in Autodock 4.2 was used, and the distinct ligand poses generated were docked into the active site of each protein. The binding energy of the ligand poses were calculated, and protein-ligand interactions were examined using Biovia DS Visualizer (Dassault Systèmes Biovia Software Inc., 2012).

### 3.7. Data Analysis

All analyzes were done in triplicate and results were gave as means ± SD. First, Principal Component Analysis (PCA) and clustered Image Maps (CIM) were applied to evaluate the effect of both key factors (Solvents and methods) on the secondary metabolites. Before doing analysis, the peak area of the secondary metabolites was log transformed. The first principal components resuming, together, a variance above 80% were selected. After, a one-way analysis of variance followed by post hoc multiple comparison tests for turkeys was achieved to assess significant differences between the extracts in terms of their chemical composition, antioxidant, and enzyme inhibitory activity, respectively (*p* < 0.05). In addition, Pearson’s correlation coefficients were calculated to analyze the relationship between secondary metabolites and antioxidant, and enzyme inhibitory activity, respectively. A Pearson’s coefficient greater than 0.7 was considered significant. Finally, Clustered Image Maps was used to evaluate the effect of solvents and methods on biological activities of the samples. For both CIMs analysis, Euclidean distance classifier and Ward’s clustering method were applied. One-way ANOVA was performed using XLSTAT (v. 2006) software environment while the correlation, PCA and CIM analysis were conducted under R (v. 3.6.1) statistical software.

## 4. Conclusions

The bioactivity, antioxidant properties, and enzyme-inhibiting effects of *A. fasciculiflora* extracts obtained from different solvents and extraction methods were investigated in this work using a variety of in vitro assays. Methanolic/Soxhlet extract had the highest TFC content, while ethyl acetate/Soxhlet extract contained the highest TPC content. However, in most antioxidant experiments, water extracts were shown to have stronger antioxidant capacity than the other extracts. Furthermore, despite the fact that different solvents and extraction methods resulted in different enzyme inhibitory effects, the results showed that most of the water extracts were inactive or weak inhibitors of the enzymes studied. In contrast, methanolic extracts were highly effective tyrosinase inhibitors. Secondary metabolites found in *A. fasciculiflora* extracts include hydroxybenzoic and hydroxycinnamic acids and derivatives, hexaric acids, coumarins, flavonoids, and hydroxycinnamic acid amides, among others. It was a fascinating look at the bioactive content and bioactivity profile of *A. fasciculiflora* based on the solvents and extraction methods used in this study that could serve as a useful reference for future research on this species.

## Figures and Tables

**Table 1:** Total phenolic and flavonoid contents and of the tested extracts.

Extraction Methods	Solvents	TPC (mg GAE/g)	TFC (mg RE/g)
MAC	EA	20.53 ± 0.12 ^b^	1.99 ± 0.26 ^gh^
	MeOH	15.34 ± 0.62 ^d^	3.26 ± 0.28 ^fgh^
	Water	18.75 ± 0.14 ^c^	4.43 ± 0.10 ^ef^
SOX	EA	24.87 ± 1.13 ^a^	2.13 ± 0.05 ^gh^
	MeOH	19.58 ± 0.23 ^bc^	25.26 ± 0.28 ^a^
	Water	23.70 ± 0.03 ^a^	6.02 ± 0.18 ^de^
HAE	EA	20.61 ± 0.45 ^b^	1.68 ± 0.23 ^h^
	MeOH	24.66 ± 0.26 ^a^	20.98 ± 1.61 ^b^
	Water	19.86 ± 0.02 ^bc^	3.55 ± 0.64 ^fg^
UAE	EA	20.51 ± 0.29 ^b^	2.44 ± 0.48 ^gh^
	MeOH	14.25 ± 0.19 ^d^	18.47 ± 0.35 ^c^
	Water	20.04 ± 0.21 ^b^	6.37 ± 0.14 ^d^

Values are reported as mean ± SD of three parallel measurements. MAC: Maceration; SOX: Soxhlet; HAE: Homogenizer assisted extraction; UAE: Ultrasound assisted extraction. TPC: Total phenolic content; TFC: Total flavonoid content; GAE: Gallic acid equivalent; RE: Rutin equivalent. Different letters in same column indicate significant differences in the tested extracts (“^a^” indicates the highest content and “^h^” indicates the lowest content, *p* < 0.05).

**Table 2:** Secondary metabolites in *Alcea fasciculiflora* extracts analysed by UHPLC-ESI/HRMS.

No. (Af)	Identified/Tentatively Annotated Compound	Molecular Formula	Exact Mass [M-H]^−^	Fragmentation Pattern in (−) ESI-MS/MS	t_R_ (Min)	Δ ppm	Distribution
**Hydroxybenzoic, hydroxycinnamic acids and derivatives**							
**1.**	gallic acid-*O*-hexoside	C_13_H_16_O_10_	331.0674	331.0674 (100), 313.0565 (6.63), 169.0130 (4.40), 16830054 (38.44), 125.0231 (35.01), 97.0281 (1.99)	1.25	2.417	2,3,5,8,11
**2.**	protocatechuic acid-*O*-hexoside	C_13_H_16_O_9_	315.0725	315.0725 (100), 153.0183 (26.65), 152.0103 (58.78), 123.0075 (2.44), 108.0202 (75.18)	1.68	1.126	2,3,5,6,8,9,11
**3.**	vanillic acid ^a^	C_8_H_8_O_4_	167.0350	167.0336 (17.07), 152.0103 (100), 123.0071 (20.77), 108.0203 (48.42), 95.0122 (4.33)	1.79	−7.196	2,4,5,8,9,11
**4.**	vanillic alcochol-*O*-hexoside	C_13_H_16_O_9_	315.0729	315.0729 (59.95), 153.0545 (100), 135.0435 (0.25), 123.0437 (53.52), 109.0280 (49.09)	2.15	2.395	1,2,3,5,6,7,8,10,11,12
**5.**	*p*-hydroxyphenylacetic acid-*O*-hexoside	C_14_H_18_O_8_	313.0938	313.0932 (100), 151.0389 (19.28), 123.0441 (4.41), 93.0329 (28.23)	2.16	2.936	2,3,5,6,8,9,11,12
**6.**	syringic acid 4-*O*-hexoside	C_15_H_20_O_10_	359.0992	359.0989 (7.49), 197.0449 (100), 182.0213 (19.70), 153.0547 (16.59), 123.0074 (33.00)	2.27	2.172	2,3,4,5,6,8,9,11,12
**7.**	caffeic acid-*O*-hexoside	C_15_H_18_O_9_	341.0881	341.0881 (4.51), 179.0341 (100), 135.0439 (58.82), 109.2430 (0.70)	2.39	0.952	2,3,4,5,6,8,9,11,12
**8.**	protocatechuic acid ^a^	C_7_H_6_O_4_	153.0183	153.0182 (16.31), 109.0280 (100), 91.0173 (1.33), 81.0331 (1.51)	2.03	−6.548	1,2,3,4,5,6,7,8,9,10,11,12
**9.**	vanillic alcohol	C_8_H_10_O_3_	153.0547	153.0545 (15.63), 137.0236 (0.46), 123.0437 (100), 109.0279 (1.08), 95.0487 (1.82)	2.14	−6.648	2,3,4,5,6,8,9,11,12
**10.**	ferulic acid-hexoside	C_16_H_20_O_9_	355.1040	355.1040 (1.0), 193.0498 (100), 178.0262 (9.5), 149.0596 (21.5), 134.0360 (57.7)	2.96	1.618	1,2,4,5,6,7,8,9,10,11,12
**11.**	salicylic acid-*O*-hexoside	C_13_H_16_O_8_	299.0774	299.0774 (100), 271.0774 (0.88), 137.0232 (20.46),	3.00	0.399	1,2,3,4,5,6,8,9,11,12
**12.**	caffeic acid ^a^	C_9_H_8_O_4_	179.0341	179.0341 (18.86), 135.0438 (100), 117.0336 (0.59), 107.0489 (1.37), 91.0538 (0.55)	3.54	−4.982	1,2,3,4,5,6,7,8,9,10,11,12
**13.**	ferulic acid-hexoside isomer	C_16_H_20_O_9_	355.1038	355.1038 (9.3), 193.0499 (100), 178.0262 (16.2), 149.0596 (12.0), 134.0360 (36.8)	3.76	0.971	1,3,4,5,6,8,9,10,11,12
**14.**	*m*-coumaric acid ^a^	C_9_H_8_O_3_	163.0390	163.0390 (7.97), 119.0488 (100), 93.0330 (0.99)	4.55	−6.608	1,2,3,4,5,6,7,8,9,10,11,12
**15.**	shikimic acid	C_7_H_10_O_5_	173.0455	173.0808 (46.78), 155.0697 (1.81), 129.0907 (5.60), 111.0800 (100)	4.93	−6.657	1,2,3,5,7,8,9,10,11,12
**16.**	ferulic acid ^a^	C_10_H_10_O_4_	193.0501	193.0499 (28.83), 178.0262 (83.59), 149.0596 (29.31), 134.0360 (100)	5.14	−2.860	2,3,4,5,6,8,9,11,12
**17.**	*o*-coumaric acid ^a^	C_9_H_8_O_3_	163.0390	163.0390 (8.41), 135.0438 (0.16), 119.0487 (100), 93.0330 (1.27)	5.55	−6.547	2,5,6,8,9,11
**18.**	salicylic acid ^a^	C_7_H_6_O_3_	137.0233	137.0231 (10.65), 108.0203 (0.51), 93.0330 (100)	6.29	−8.519	1,2,3,4,5,6,7,8,9,10,11,12
**Hexaric acids**							
**19.**	*p*-coumaroylhexaric acid	C_15_H_16_O_10_	355.0674	355.0674 (6.2), 209.0298 (22.9), 191.0190 (40.2), 163.0389 (3.6), 147.0286 (16.2), 129.0181 (9.5), 119.0488 (2.3), 111.0073 (5.1), 85.0279 (100)	2.42	1.042	2,3,5,6,8,9,11,12
**20.**	*p*-coumaroylhexaric acid isomer	C_15_H_16_O_10_	355.0677	355.0677 (6.6), 209.0296 (23.6), 191.0189 (40.5), 163.0391 (3.0), 147.0287 (16.0), 129.0179 (10.0), 119.0488 (2.0), 111.0074 (4.5), 85.0279 (100)	2.42	1.747	2,3,5,6,9,11,12
**21.**	*p*-coumaroylhexaric acid isomer	C_15_H_16_O_10_	355.0677	355.0677 (5.1), 209.0297 (25.1), 191.0190 (40.7), 163.0392 (3.8), 147.0287 (16.6), 129.0177 (7.7), 119.0488 (1.6), 111.0074 (4.1), 85.0279 (100)	3.15	−1.182	1,2,3,4,5,6,8,9,11,12
**22.**	feruloylhexaric acid	C_16_H_18_O_11_	385.0784	385.0784 (10.1), 209.0297 (9.9), 193.0500 (3.2), 191.0189 (51.0), 147.0284 (17.2), 134.0360 (2.9), 129.0180 (10.7), 111.0072 (3.3), 85.0279 (100)	2.96	1.988	1,2,3,5,6,8,9,11,12
**23.**	feruloylhexaric acid isomer	C_16_H_18_O_11_	385.0774	385.0774 (7.1), 209.0294 (9.5), 193.0501 (2.3), 191.0190 (54.1), 147.0287 (18.9), 134.0362 (3.2), 129.0180 (10.5), 111.0074 (3.6), 85.0279 (100)	3.18	−0.635	2,3,5,6,8,9,12
**24.**	feruloylhexaric acid isomer	C_16_H_18_O_11_	385.0771	385.0771 (12.9), 209.0296 (12.5), 193.0500 (2.9), 191.0190 (50.6), 149.0232 (12.5), 134.0359 (2.8), 129.0180 (11.4), 85.0279 (100)	3.38	−1.386	2,3,5,6,8,9,11,12
**25.**	feruloylhexaric acid isomer	C_16_H_18_O_11_	385.0778	385.0778 (8.6), 209.0296 (10.9), 193.0500 (1.9), 191.0189 (57.6), 147.0287 (15.2), 134.0361 (4.6), 111.0073 (4.8), 85.0279 (100)	3.61	0.482	2,3,5,6,8,9,11,12
**Coumarins**							
**26.**	aesculin	C_15_H_16_O_9_	339.0729	339.0724 (23.13), 177.0183 (100), 149.0233 (1.33), 133.0281 (10.44), 105.0331 (3.72), 89.0381 (2.24)	2.69	2.137	1,2,4,6,10,11
**27.**	aesculetin	C_9_H_6_O_4_	177.0186	177.0184 (100), 149.0233 (3.62), 133.0281 (21.03), 121.0281 (0.94), 105.0331 (10.06), 89.0381 (8.06)	3.45	−4.191	1,2,3,4,5,6,7,8,9,10,11,12
**28.**	fraxin	C_16_H_16_O_10_	369.0832	369.0830 (15.63), 207.0292 (100), 192.0056 (49.59), 164.0103 (1.53), 123.0073 (1.42)	3.52	1.192	1,2,3,5,6,8,10,11,12
**29.**	umbelliferone	C_9_H_6_O_3_	161.0234	161.0233 (77.03), 133.0281 (100), 115.0178 (1.47), 105.0332 (2.02), 89.0379 (1.09)	3.78	−6.442	2,3,5,6,8,9,11,12
**30.**	fraxetin	C_10_H_8_O_5_	207.0294	207.0292 (28.75), 192.0055 (100), 164.0103 (3.60), 123.0075 (2.94)	4.22	−2.544	1,2,3,4,8,9,10,11
**31.**	coumarin	C_9_H_6_O_2_	145.0284	145.0283 (35.47), 117.0332 (100), 102.0545 (5.52), 89.0381 (1.73)	4.52	−7.604	1,2,3,5,6,7,9,10,11,12
**32.**	scopoletin	C_10_H_8_O_4_	191.0343	191.0342 (19.51), 176.0105 (100), 148.0154 (15.95), 104.0253 (18.79)	5.05	−3.465	1,2,3,4,5,6,7,8,9,10,11,12
**Flavonoids**							
**33.**	myricetin-3-*O*-rutinoside	C_27_H_30_O_17_	625.1418	625.1418 (100), 317.0289 (18.42), 316.0226 (81.70), 287.0200 (16.61), 271.0251 (25.56), 178.9976 (4.46), 157.0022 (4.21), 137.0231 (0.78), 107.0127 (0.67)	4.48	1.324	2,5,8,11
**34.**	myricetin-3-*O*-hexoside	C_21_H_20_O_13_	479.0839	479.0835 (100), 317.0297 (18.03), 316.0228 (89.81), 287.0201 (15.13), 271.0248 (21.32), 178.9977 (1.63), 151.0018 (4.89)	4.58	1.683	2,5,8,11
**35.**	isorhamnetin 3-*O*-rutinoside ^a^	C_26_H_2__8_O_15_	623.1618	623.1625 (100), 315.0492 (8.44), 314.0436 (70.10), 300.0261 (8.99), 299.0200 (54.91), 271.0251 (26.91), 255.0299 (3.40), 243.0297 (5.02), 227.0347 (3.24), 151.0024 (0.25)	5.13	0.758	2,5,11,12
**36.**	patuletin-3-*O*-glucoside	C_2__2_H_2__2_O_1__3_	493.0997	493.0995 (100), 331.0416 (15.90), 330.0384 (48.73), 316.0223 (22.3), 315.0151 (33.33), 287.0205 (12.67), 271.0253 (4.11), 259.0246 (2.94), 243.0296 (3.30), 178.9979 (0.79), 139.0024 (2.11), 136.9866 (0.72), 151.0008 (0.45), 121.0275 (0.58)	5.26	1.959	2,3,5,12
**37.**	isoquercitrin ^a^	C_21_H_20_O_12_	463.0888	463.0888 (100), 301.0351 (41.40), 300.0277 (77.04), 271.0249 (36.72), 255.0298 (16.35), 227.0347 (2.25), 151.0023 (5.9), 121.0248 (1.25), 107.0124 (2.58)	5.29	1.254	2,5,6,8,11
**38.**	kaempferol-3-*O*-rutinoside ^a^	C_2__7_H_30_O_1__5_	593.1522	593.1518 (100), 285.0404 (99.85), 284.0327 (57.50), 255.0298 (42.81), 227.0347 (28.43), 151.0024 (2.61), 135.0078 (1.02), 107.0121 (2.54)	5.64	−0.107	1,2,3,4,5,7,8,9,10,11,12
**39.**	kaempferol-3-*O*-glucoside ^a^	C_21_H_20_O_11_	447.0941	447.0938 (100), 285.0399 (23.82), 284.0329 (58.22), 255.0250 (36.72), 227.0347 (2.25), 151.0025 (2.12), 107.0125 (0.79)	5.85	−0.145	1,2,3,4,5,6,7,8,9,10,11,12
**40.**	quercetin-*O*-hexuronide	C_2__1_H_18_O_13_	477.0677	477.0677 (100), 301.0356 (100), 300.0273 (2.57), 227.0343 (2.11), 151.0032 (0.52), 107.0126 (0.34)	6.85	0.600	2,5,11
**41.**	luteolin ^a^	C_15_H_10_O_6_	285.0410	285.0406 (100), 151.0024 (4.29), 149.0229 (0.29), 133.0282 (22.21), 121.0281 (0.80), 107.0124 (3.33)	7.57	2.065	1,2,4,5,7,8,10,11
**42.**	quercetin ^a^	C_15_H_10_O_7_	301.0356	301.0356 (100), 273.0399 (0.97), 257.0470 (5.93), 178.9976 (22.06), 151.0025 (43.82), 149.0239 (1.38) 121.0281 (14.69), 107.0122 (12.18)	7.63	0.578	2,3,5,9,12
**43.**	tiliroside ^a^	C_30_H_26_O_13_	593.1301	593.1307 (100), 447.0959 (1.89), 285.0404 (79.06), 284.0327 (56.62), 255.0298 (38.90), 227.0345 (28.83), 151.0025 (4.18), 107.0123 (2.48), 135.0073 (1.59)	7.68	0.010	1,2,4,5,7,8,10,11,12
**44.**	kaempferol-*O*-*p*-coumaroyl-*O*-hexoside	C_30_H_26_O_13_	593.1308	593.1307 (100), 447.0925 (2.51), 285.0404 (76.82), 284.0328 (61.03), 255.0298 (41.36), 227.0345 (28.72), 151.0021 (3.15), 107.0124 (2.27), 135.0077 (1.58)	7.94	1.241	1,2,4,5,7,8,10,11,12
**45.**	naringenin	C_15_H_12_O_5_	271.0615	271.0615 (100), 151.0025 (68.67), 119.0488 (55.13), 107.0123 (18.70)	8.56	1.082	2
**46.**	apigenin ^a^	C_15_H_10_O_5_	269.0456	269.0456 (100), 225.0549 (1.28), 151.0024 (4.59), 149.0232 (5.01), 117.0331 (17.57), 107.0123 (5.21)	8.63	0.273	1,2,4,5,7,8,10,11
**47.**	kaempferol ^a^	C_15_H_10_O_6_	285.0409	285.0405 (100), 227.0348 (0.48), 151.0026 (1.69), 117.0331 (0.78), 107.0123 (0.82)135.0074 (0.4), 107.0124 (1.4)	8.80	0.136	2,4,5,8,10,11
**Hydroxycinnamic acid amides**							
**No.**	**Identified/tentatively annotated compound**	**Molecular formula**	**Exact mass** **[M + H]^+^**	**Fragmentation pattern in** ** (+) ** **ESI-MS/MS**	**t_R_** **(min)**	**Δ ppm**	**Distribution**
**48.**	N-coumaroyl tyramine	C_17_H_17_ O_3_N	284.1287	284.1275 (24.8), 147.0439 (100), 139.0908 (0.7), 121.0649 (38.0), 119.0493 (15.8), 103.0546 (6.5)	6.95	−2.076	2,4,5,8,11
**49.**	N-feruloyl tyramine	C_18_H_19_O_4_N	314.1377	314.1380 (46.4), 177.0545 (100), 149.0596 (5.0), 145.0283 (30.9), 121.0649 (43.6), 103.0546 (7.9)	7.22	−2.179	1,2,3,4,5,6,7,8,9,10,11,12
**50.**	N-feruloyl-3-methoxytyramine	C_19_H_21_O_5_N	344.1489	344.1487 (8.5), 177.0544 (100), 149.0596 (4.5), 145.0282 (29.9), 117.0336 (17.4)	7.51	−0.298	2,5

^a^—compare to reference standard. 1. MAC-EA, 2. MAC-MeOH, 3. MAC-Water, 4. SOX-EA, 5. SOX-MeOH, 6. SOX-Water, 7. HAE-EA, 8. HAE-MeOH, 9. HAE-Water, 10. UAE-EA, 11. UAE-MeOH, 12. UAE-Water. Δ (ppm)—a mass error in ppm of the assignment, when comparing the exact *m*/*z* of [M-H]^−^ and the accurate *m*/*z*.

**Table 3:** Free radical scavenging abilities, reducing power, metal chelating and total antioxidant (by phosphomolybdenum assay) abilities of the tested extracts.

Extraction Methods	Solvents	DPPH (mg TE/g)	ABTS (mg TE/g)	CUPRAC (mg TE/g)	FRAP (mg TE/g)	MCA (mg EDTAE/g)	PBD (mmol TE/g)
MAC	EA	4.86 ± 0.51 ^gh^	21.40 ± 1.79 ^g^	56.11 ± 0.21 ^d^	19.53 ± 0.70 ^f^	18.28 ± 1.57 ^g^	1.88 ± 0.08 ^abc^
	MeOH	7.26 ± 0.09 ^f^	31.26 ± 0.44 ^e^	72.89 ± 1.50 ^b^	24.35 ± 0.77 ^e^	26.78 ± 0.74 ^de^	1.67 ± 0.11 ^c^
	Water	17.65 ± 0.28 ^d^	54.18 ± 1.03 ^d^	42.26 ± 0.30 ^f^	28.21 ± 0.26 ^d^	33.54 ± 1.04 ^c^	1.07 ± 0.03 ^de^
SOX	EA	3.37 ± 0.20 ^h^	24.84 ± 0.37 ^f^	64.13 ± 4.74 ^c^	23.75 ± 0.46 ^e^	32.54 ± 0.77 ^c^	1.98 ± 0.05 ^a^
	MeOH	26.75 ± 1.40 ^b^	58.19 ± 1.71 ^c^	62.26 ± 1.12 ^c^	35.20 ± 1.03 ^b^	29.09 ± 2.39 ^d^	1.68 ± 0.03 ^bc^
	Water	35.33 ± 0.71 ^a^	76.27 ± 1.13 ^a^	64.13 ± 0.10 ^c^	42.58 ± 0.07 ^a^	45.67 ± 0.17 ^a^	0.96 ± 0.02 ^e^
HAE	EA	2.63 ± 0.16 ^h^	14.45 ± 0.76 ^h^	61.00 ± 1.87 ^cd^	23.06 ± 0.62 ^e^	19.97 ± 0.97 ^g^	2.07 ± 0.18 ^a^
	MeOH	7.15 ± 0.92 ^fg^	23.21 ± 0.80 ^fg^	78.32 ± 2.26 ^a^	17.51 ± 0.29 ^g^	40.05 ± 1.12 ^b^	1.93 ± 0.13 ^ab^
	Water	20.54 ± 1.87 ^c^	64.02 ± 0.85 ^b^	49.12 ± 0.33 ^e^	31.52 ± 0.63 ^c^	46.71 ± 0.20 ^a^	0.90 ± 0.03 ^e^
UAE	EA	2.66 ± 0.07 ^h^	13.46 ± 0.65 ^h^	64.22 ± 1.58 ^c^	24.37 ± 0.93 ^e^	23.56 ± 0.51 ^ef^	2.12 ± 0.12 ^a^
	MeOH	13.49 ± 0.15 ^e^	33.15 ± 0.45 ^e^	40.38 ± 1.25 ^f^	22.77 ± 0.72 ^e^	20.74 ± 1.25 ^fg^	1.25 ± 0.05 ^d^
	Water	26.80 ± 0.61 ^b^	58.70 ± 1.05 ^c^	50.55 ± 0.36 ^e^	35.09 ± 0.80 ^b^	40.76 ± 0.25 ^b^	1.03 ± 0.01 ^de^

Values are reported as mean ± SD of three parallel measurements. MAC: Maceration; SOX: Soxhlet; HAE: Homogenizer assisted extraction; UAE: Ultrasound assisted extraction. TE: Trolox equivalent; EDTAE: EDTA equivalent. TE: Trolox equivalent. Different letters in same column indicate significant differences in the tested extracts (“^a^” indicates the strongest ability and “^h^” indicates the weakest ability, *p* < 0.05).

**Table 4:** Enzyme inhibitory effects of the tested extracts.

Extraction Methods	Solvents	AChE (mg GALAE/g)	BChE (mg GALAE/g)	Tyrosinase (mg KAE/g)	Amylase (mmol ACAE/g)	Glucosidase (mmol ACAE
MAC	EA	2.44 ± 0.42 ^a^	4.61 ± 1.08 ^ab^	1.79 ± 0.16 ^e^	1.03 ± 0.06 ^ab^	0.73 ± 0.06 ^a^
	MeOH	2.45 ± 0.19 ^a^	4.63 ± 0.23 ^ab^	58.93 ± 0.62 ^a^	0.65 ± 0.03 ^c^	0.56 ± 0.02 ^b^
	Water	0.33 ± 0.03 ^b^	na	16.15 ± 1.21 ^d^	0.14 ± 0.01 ^e^	na
SOX	EA	2.58 ± 0.61 ^a^	3.04 ± 0.16 ^c^	30.26 ± 1.59 ^c^	1.07 ± 0.03 ^ab^	0.74 ± 0.01 ^a^
	MeOH	2.17 ± 0.05 ^a^	1.17 ± 0.19 ^d^	54.44 ± 0.15 ^b^	0.50 ± 0.01 ^d^	na
	Water	0.21 ± 0.01 ^b^	na	18.12 ± 1.66 ^d^	0.12 ± 0.01 ^e^	na
HAE	EA	2.33 ± 0.36 ^a^	4.09 ± 0.20 ^bc^	33.36 ± 3.20 ^c^	0.99 ± 0.03 ^b^	0.76 ± 0.04 ^a^
	MeOH	2.23 ± 0.06 ^a^	5.80 ± 1.22 ^a^	57.23 ± 0.82 ^ab^	1.02 ± 0.04 ^ab^	0.11 ± 0.03 ^c^
	Water	0.17 ± 0.02 ^b^	na	17.28 ± 1.04 ^d^	0.14 ± 0.01 ^e^	na
UAE	EA	2.80 ± 0.27 ^a^	na	17.85 ± 1.08 ^d^	1.11 ± 0.05 ^a^	0.73 ± 0.02 ^a^
	MeOH	2.58 ± 0.13 ^a^	1.23 ± 0.11 ^d^	53.43 ± 0.67 ^b^	0.55 ± 0.02 ^d^	na
	Water	0.39 ± 0.09 ^b^	na	17.73 ± 1.65 ^d^	0.11 ± 0.01 ^e^	na

Values are reported as mean ± SD of three parallel measurements. MAC: Maceration; SOX: Soxhlet; HAE: Homogenizer assisted extraction; UAE: Ultrasound assisted extraction. GALAE: Galantamine equivalent; KAE: Kojic acid equivalent; ACAE: Acarbose equivalent; na: not active. Different letters in same column indicate significant differences in the tested extracts (“^a^” indicates the strongest ability and “^e^” indicates the weakest ability, *p* < 0.05).

**Table 5:** Calculated binding affinity of bioactive constituents from secondary metabolites in *Alcea fasciculiflora* extracts.

S/N	Compound	AChE	BChE	Tyrosinase	Amylase	Glucosidase
		Kcal/moL				
**11**	Salicylic acid-O-hexoside	−9.43	−10.53	−9.48	−6.96	−10.05
**16**	Ferulic acid	−6.25	−8.31	−8.80	−3.76	−6.76
**18**	Salicylic acid	−5.57	−7.70	−8.60	−3.08	−4.41
**31**	Coumarin	−7.90	−4.65	−2.69	−3.62	−6.32
**38**	Kaempferol-3-rutinoside	−11.42	−11.92	−7.50	−9.95	−10.65
**39**	Kaempferol-3-glucoside	−10.22	−9.60	−5.61	−7.57	−8.50
**43**	Tiliroside	−13.91	−10.63	−5.64	−6.99	−4.55
**44**	Kaempferol-O-p-coumaroyl-O-hexoside	−11.35	−7.34	−5.72	−3.51	−5.66
**46**	Apigenin	−9.98	−7.34	−4.29	−6.22	−7.06
**49**	N-feruloyl tyramine	−10.53	−9.56	−5.94	−7.67	−10.25

## Data Availability

Not applicable.

## Supplementary Materials

The following supporting information can be downloaded at: https://www.mdpi.com/article/10.3390/molecules27155011/s1, Table S1. Percentage of variability and eigenvalue explained by each principal component of Principal Component Analysis. Figure S1. Relation between the bioactivities and the first four retained principal component. For compounds numbers refer to Table 1.
