# Supplementary material for: Which Extraction Solvents and Methods Are More Effective in Terms of Chemical Composition and Biological Activity of Alcea fasciculiflora from Turkey?
Source: Molecules. 2022 Aug 6;27(15):5011. doi: 10.3390/molecules27155011 (PMC9370370; doi:10.3390/molecules27155011)
Supplement: Supplementary file 1 [file molecules-27-05011-s001.zip › molecules-1851014-supplementary.pdf]

Article

Which Extraction Solvents and Methods Are More Effective in Terms of Chemical Composition and Biological Activity of *Alcea fasciculiflora* from Turkey?

Refiye Beyza Ozturk [1], Gokhan Zengin [1,*], Kouadio Ibrahime Sinan [1], Domenico Montesano [2,*], Dimitrina Zheleva-Dimitrova [3], Reneta Gevrenova [3], Abdullahi Ibrahim Uba [4], Ugur Cakilcioglu [5], Alevcan Kaplan [6], Sharmeen Jugreet [7], Stefano Dall'Acqua [8] and Mohamad Fawzi Mahomoodally [7]

[1] Physiology and Biochemistry Research Laboratory, Department of Biology, Science Faculty, Selcuk University, Konya 42130, Turkey
[2] Department of Pharmacy, University of Naples Federico II, Via D. Montesano 49, 80131 Naples, Italy
[3] Department of Pharmacognosy, Faculty of Pharmacy, Medical University-Sofia, 1431 Sofia, Bulgaria
[4] Department of Molecular Biology and Genetics, Faculty of Engineering and Natural Sciences, Kadir Has University, Istanbul 34083, Turkey
[5] Pertek Sakine Genç Vocational School, Munzur University, 62500 Tunceli, Turkey
[6] Sason Vocational School, Batman University, Batman 72100, Turkey
[7] Department of Health Sciences, Faculty of Medicine and Health Sciences, University of Mauritius, 80837 Réduit, Mauritius
[8] Department of Pharmaceutical and Pharmacological Sciences, University of Padova, Via Marzolo 5, 35131 Padova, Italy
* Correspondence: gokhanzengin@selcuk.edu.tr (G.Z.); domenico.montesano@unina.it (D.M.)

Table S1. Percentage of variability and eigenvalue explained by each principal component of Principal Component Analysis.

Principal components	Eigenvalue	Proportion of explained variance	Cumulative proportion of explained variance
**PC1**	22,09	44,19	44,19
**PC2**	12,74	25,49	69,68
**PC3**	3,65	7,30	76,98
**PC4**	2,64	5,28	82,26
**PC5**	2,26	4,52	86,79
**PC6**	1,98	3,97	90,76

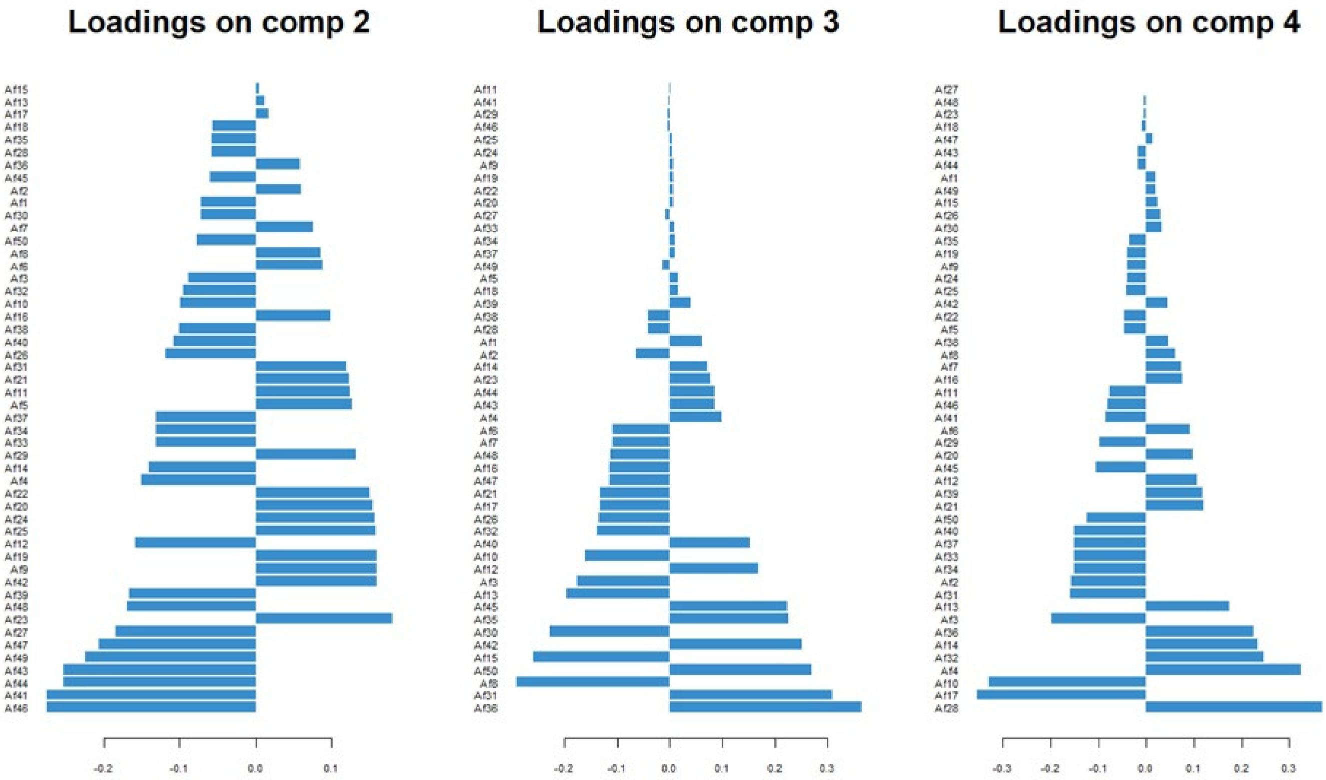

Figure S1. Relation between the bioactivities and the first four retained principal component. For compounds numbers refer to Table 1.
